# Exercise training and endothelial function in patients with type 2 diabetes: a meta-analysis

**DOI:** 10.1186/s12933-018-0711-2

**Published:** 2018-05-02

**Authors:** Shanhu Qiu, Xue Cai, Han Yin, Zilin Sun, Martina Zügel, Jürgen Michael Steinacker, Uwe Schumann

**Affiliations:** 1 Department of Endocrinology, Zhongda Hospital, Institute of Diabetes, School of Medicine, Southeast University, Nanjing, People’s Republic of China; 2 Division of Sports and Rehabilitation Medicine, Ulm University Medical Center, Ulm, Germany

**Keywords:** Exercise training, Endothelial function, Flow-mediated dilation, Type 2 diabetes

## Abstract

**Background and aims:**

Exercise training is considered a cornerstone in the management of type 2 diabetes, which is associated with impaired endothelial function. However, the association of exercise training with endothelial function in type 2 diabetes patients has not been fully understood. This meta-analysis aimed to investigate their associations with focus on exercise types.

**Methods:**

Databases were searched up to January 2018 for studies evaluating the influences of exercise training with durations ≥ 8 weeks on endothelial function assessed by flow-mediated dilation (FMD) among type 2 diabetes patients or between type 2 diabetics and non-diabetics. Data were pooled using random-effects models to obtain the weighted mean differences (WMDs) and 95% confidence intervals (CIs).

**Results:**

Sixteen databases were included. Exercise training resulted in an overall improvement in FMD by 1.77% (95% CI 0.94–2.59%) in type 2 diabetes patients. Specifically, both aerobic and combined aerobic and resistance exercise increased FMD by 1.21% (95% CI 0.23–2.19%) and 2.49% (95% CI 1.17–3.81%), respectively; but resistance exercise only showed a trend. High-intensity interval aerobic exercise did not significantly improve FMD over moderate-intensity continuous exercise. Notably, the improvement in FMD among type 2 diabetes patients was smaller compared with non-diabetics in response to exercise training (WMD − 0.72%, 95% CI − 1.36 to − 0.08%) or specifically to aerobic exercise (WMD − 0.65%, 95% CI − 1.31 to 0.01%).

**Conclusions:**

Exercise training, in particular aerobic and combined exercise, improves endothelial function in type 2 diabetes patients, but such an improvement appears to be weakened compared with non-diabetics.

*Trial registration* PROSPERO CRD42018087376

**Electronic supplementary material:**

The online version of this article (10.1186/s12933-018-0711-2) contains supplementary material, which is available to authorized users.

## Introduction

The endothelium, a monolayer of cells that provides a physical barrier between vessel lumen and vascular wall, is essential in maintaining vascular homeostasis, a process which is recognized to be primarily modulated via its release of a list of mediators that regulate blood coagulation and vascular tone [1–3]. Endothelial dysfunction is referred to the condition where the endothelium loses its physiological properties but shows a tendency towards vasoconstriction, pro-thrombotic, and pro-inflammatory states [2, 3]. In addition to being a well-recognized precursor of atherosclerosis [4, 5], endothelial dysfunction has also been considered a pathophysiological hallmark characterized by type 2 diabetes [3]. This originates in the evidence that endothelial dysfunction is consistently observed in patients with type 2 diabetes [5–7] and predicts the risk of incident type 2 diabetes [8]. On the other hand, endothelial dysfunction is recognized to be an initiating and important factor in the development and progression of diabetes related microvascular and macrovascular complications [5, 9].

Since exercise training is a key element in the management of type 2 diabetes [10–14], and given that endothelial dysfunction might be a therapeutic target for diabetes [14, 15], there is a growing interest in exploring the influence of exercise training on endothelial function in patients with type 2 diabetes [16–24]. However, available studies on this topic have shown inconsistent and inconclusive findings. Some randomized controlled trials (RCTs) have indicated that exercise training improves endothelial function, while others noted that it may not. Moreover, most of these studies had small sample sizes [17–19, 21–23], ranging from 13 to 39. Noteworthy, Montero and colleagues conducted a meta-analysis with enhanced statistical power in 2013 pointing out that in patients with type 2 diabetes exercise training increased flow-mediated dilation (FMD)—a non-invasive but the most widely used approach for endothelial function assessment [25], but their conclusion was based on five RCTs from four articles [26] while more related RCTs were published since then [18, 22, 24]. Furthermore, the authors did not assess the influences of different exercise training types (e.g., aerobic, resistance, or combined training) on endothelial function, nor explored the potential moderators (e.g., glycemic control, blood pressure, or cardiorespiratory fitness) in predicting the changes in endothelial function related to exercise training, possibly because of the limited number of studies available at that time.

Therefore, we conducted this meta-analysis by incorporating the latest evidence with a primary focus on the impacts of exercise training and exercise types on endothelial function assessed by FMD in patients with type 2 diabetes as well as on the investigation of their potential moderators. Moreover, since endothelial function is evidentially impaired in patients with type 2 diabetes compared with non-diabetic controls [5, 6], our secondary aim was to assess whether the presence of type 2 diabetes would attenuate the improvement in endothelial function in response to exercise training.

## Methods

This meta-analysis is reported following the Preferred Reporting Items for Systematic Reviews and Meta-Analyses (PRISMA) guideline [27], with its protocol registered in PROPERO as CRD42018087376.

### Search strategy and study selection

A systematic literature search for relevant studies published in English was conducted in the databases of PubMed, the Cochrane Central Register of Controlled Trials, and Web of Science from their inceptions to January 12nd, 2018. In addition, the reference lists of relevant articles, reviews, and meta-analyses were manually checked for other suitable studies. The words or terms used for searching were linked with “endothelial function”, “diabetes”, and “exercise training” (see Additional file 1: Table S1).

Studies eligible for inclusion in this meta-analysis required to fulfill the following criteria: (i) participants were diagnosed with type 2 diabetes; (ii) the intervention groups received land-based normoxic exercise training programs with durations ≥ 8 weeks, a time-window which is commonly employed to assess the chronic exercise training effects on health outcomes [28]; (iii) the controls received no exercise (or usual care), exercise training programs different from the intervention groups, or non-exercise intervention programs comparable to the intervention groups; (iv) outcomes on endothelial function assessed by FMD had to be reported pre- and post-intervention; and (v) allocation to the intervention or control group should be random. Studies were also considered eligible if they compared the effects of exercise training with durations ≥ 8 weeks on endothelial function assessed by FMD in patients with type 2 diabetes versus non-diabetes controls. Studies were excluded if they were posters or protocols, had a lack of proper controls, or did not report outcomes on FMD.

Two authors (S.Q. and X.C.) reviewed the titles, abstracts, and/or full-texts for each of the articles identified by the literature search after removal of duplicates, aiming to determine the eligibility for this meta-analysis. During the study selection process, discrepancies were resolved by discussion with a third author (U.S. or Z.S).

### Data extraction and quality assessment

A standardized data abstraction form was applied to collect the following information from eligible studies: author information, participant characteristics [including sex distribution (proportion of males), duration of diabetes, and the means of age, body mass index (BMI), blood pressure, glycemic control, and cardiorespiratory fitness at baseline], intervention details (including type of exercise, intensity, time for one bout of exercise, frequency, and intervention duration), and study outcomes (including FMD at baseline and post-intervention). If the outcomes of interest were incomplete or could not be imputed, corresponding authors of the original studies were contacted via emails. Moreover, in this meta-analysis FMD was calculated as the percent change in diameters following reactive hyperemia compared with the baseline diameters at rest.

The Cochrane Collaboration “Risk of Bias” tool [29], which includes items on selection bias, performance bias, detection bias, attrition bias, and reporting bias, was applied to evaluate the quality of eligible studies. All the data collection and quality assessment were initially done by one author (S.Q.), and later checked by another author (X.C.). Discrepancies, if occurred, were resolved by referring back to the original studies.

### Data synthesis and statistical analysis

For studies reporting standard errors, 95% confidence intervals (CIs), or interquartile ranges, the standard deviations were obtained using the methods described in Cochrane Handbook for Systematic Reviews [29] or reported previously [30]. For studies including two different exercise training interventions, the control group was split into two groups with smaller sample sizes, aiming to provide reasonably independent comparisons and to overcome the unit-of-analysis error [29]. Post-intervention FMD values were primarily chosen for analysis in general, but only the change scores from baseline were selected for assessing the impact of the existence of type 2 diabetes on endothelial function in response to exercise training, which is because the baseline FMD results were not comparable between type 2 diabetes patients and non-diabetes controls.

The weighted mean differences (WMDs) with 95% CIs were calculated using a random-effects model, which seems to better account for between-study heterogeneity and could provide more conservative results than a fixed-effects model [29]. The heterogeneity was evaluated using the *I*^*2*^ statistic, with the value > 50% indicative of significant heterogeneity [29]. Subgroup analysis was conducted to investigate the impact of exercise types on endothelial function, and meta-regression analyses were undertaken to assess the influence of patient and intervention characteristics in moderating changes in endothelial function. Sensitivity analyses were performed to assess the robustness of the findings by restricting the analyses to studies using exercise training as the sole intervention, reporting no or only minor changes in medication use during the intervention periods, or employing the intention-to-treat analysis. Publication bias was evaluated using the Begg’s and Egger’s tests, with the *P* < 0.10 indicative of significance [29]. All the analyses were conducted using STATA software (Version 12.0, StataCorp LP, College Station, Texas). A 2-sided *P* < 0.05 was considered statistically significant unless otherwise indicated.

## Results

### Literature search and study characteristics

**Fig. 1:**
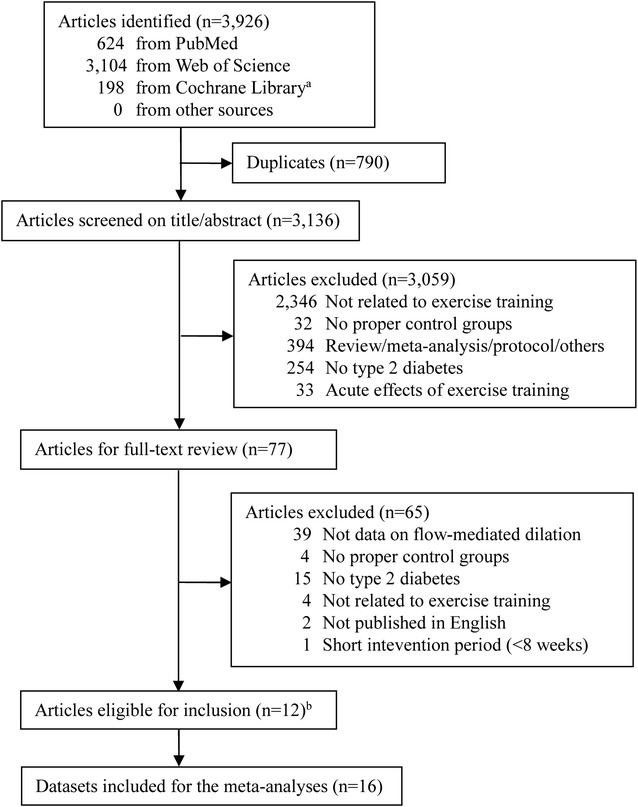
Literature search and study selection. ^a^ The database of the Cochrane Central Register of Controlled Trials was chosen. ^b^ Two studies allowed for three independent comparisons for each [17, 18]

The literature search result and study selection process are shown in Fig. 1. Of the 3136 unique articles identified, 77 were searched for full-text assessment after screening of titles and/or abstracts, with 12 considered eligible for inclusion [16–24, 31–33]. Since two articles had two different exercise training protocols with a non-exercise control group, providing three independent comparisons for each [17, 18], a total of 16 studies (databases) were finally included.

The main characteristics of participants and exercise interventions are presented in Table 1. There were 477 participants enrolled in the 16 studies, of which 13 explored the effects of exercise training on endothelial function in type 2 diabetes patients, and the remaining three assessed the influence of the presence of type 2 diabetes on the improvement in endothelial function related to exercise training. Among studies with available information, the mean age of type 2 diabetes patients was 54.2 years, the BMI was 30.0 kg/m^2^, and the duration of diabetes was 8.9 years. None of the included studies provided information on creatinine levels or estimated glomerular filtration rates, but nearly half of them had indicated the exclusion of patients with chronic kidney diseases [16, 18, 19, 21, 23, 24]. There were no changes in medication use throughout the intervention periods in most studies that assessed exercise training effects on endothelial function in type 2 diabetes patients [16–18, 21–23].

**Table 1:** Characteristics of studies included in this meta-analysis

Study, country	Age^a^, year	BMI^a^, kg/m^2^	Descriptions of intervention and control groups	Duration, weeks	Cuff pressure, mmHg	Results on FMD^b^
A. Exercise training and endothelial function in type 2 diabetes						
(i) Aerobic exercise						
Choi et al. 2012 [16]; Korea	53.8	26.8 in average	Intervention: 60 min/session of walking at moderate intensity, 5 times/week	12	250 mmHg	No change
	55.0	26.8 in average	Control: maintained usual activities and were required not to exercise			
Kwon et al. [17]; Korea^c^	55.5	26.7	Intervention: 60 min/session of walking at moderate intensity, 5 times/week	12	250 mmHg	Increase
	58.9	27	Control: maintained usual activities and were required not to exercise			
Mitranun et al. [18]; Thailand^c^	61.2	29.6	Intervention: 20–30 min/session of walking or running consisted of 4–6 intervals of 1 min exercise at 80–85% VO_2peak_ with a 4 min exercise at 50–60% VO_2peak_, 3 times/week	12	50 mmHg over SBP	Increase
	60.9	29.7	Control: maintained sedentary as previous			
Mitranun et al. [18]; Thailand^c^	61.7	29.4	Intervention: 20–30 min/session of walking or running at 60–65% VO_2peak_, 3 times/week	12	50 mmHg over SBP	Increase
	60.9	29.7	Control: maintained sedentary as previous			
Wycherley et al. 2008 [19]; Australia	51.7	33.6	Intervention: 25–60 min/session of walking or jogging at intensity increasing from 60 to 80% HR_max_, 4–5 times/week, plus a moderate energy-restricted dietary programme	12	200 mmHg	No change
	53.0	34.6	Control: a moderate energy-restricted dietary program as intervention			
(ii) Aerobic combined with resistance exercise						
Gibbs et al. 2012 [20]; USA	58	32.3	Intervention: 45 min of aerobic exercise at 60–90% HR_max_, along with 7 weight training exercises with two sets of 12–15 repetitions at 50% of 1-repetition maximum for each session, 3 times/week, plus usual care	26	> 200 mmHg	No change
	56	33.5	Control: usual care			
Maiorana et al. 2001 [21]; Australia	52	NS	Intervention: 60 min/session (15 exercises) of combined aerobic exercise (riding/walking) at 70–85% HR_max_ and resistance training at 55–65% MVC, with training intensity and duration gradually increased, 3 times/week,	8	250 mmHg	Increase
	52	NS	Control: were not required to exercise			
Naylor et al. 2016 [22]; Australia	17.3	36.1	Intervention: 60 min/session of combined aerobic exercise at 65–85% HR_max_ and resistance training at 55–70% MVC, with training volume gradually increased, 3 times/week, plus standard care	12	220 mmHg	No change
	15.3	30.0	Control: standard care			
Okada et al. 2010 [23]; Japan	61.9	25.7	Intervention: 20 min of aerobic dancing, 20 min of bicycle riding, and 20 min of resistance training for each session, 3–5 times/week, plus usual care	12	220 mmHg	No change
	64.5	24.5	Control: usual care			
(iii) Resistance exercise						
Kwon et al. [17]; Korea^c^	56.3	27.4	Intervention: 40 min of resistance bands exercise at gradually increased intensities, three sets of 10–15 exercises for each session, 3 times/week	12	250 mmHg	No change
	58.9	27	Control: maintained usual activities and were not required to exercise			
(iv) Interval versus continuous aerobic exercise						
Hollekim-Strand et al. 2014 [24]; Norway	58.6	30.2	Interval: 25 min/session of walking or jogging consisted of four intervals of 4 min exercise at 90–95% HR_max_ with a 3 min exercise at 70% HR_max_, 3 times/week	12	NS	Increase
	54.7	29.7	Continuous: 210 min/week of home-based moderate intensity exercise			
Mitranun et al. [18]; Thailand^c^	61.2	29.6	Interval: 20–30 min/session of walking or running consisted of 4–6 intervals of 1 min exercise at 80–85% VO_2peak_ with a 4 min exercise at 50–60% VO_2peak_, 3 times/week	12	50 mmHg over SBP	No change
	61.7	29.4	Continuous: 20–30 min/session of walking or running at 60–65% VO_2peak_, 3 times/week			
(v) Aerobic versus resistance exercise						
Kwon et al. [17]; Korea^c^	55.5	26.7	Aerobic: 60 min/session of walking at moderate intensity, 5 times/week	12	250 mmHg	No change
	56.3	27.4	Resistance: 40 min of resistance bands exercise at gradually increased intensities, 3 sets of 10–15 exercises for each session, 3 times/week			
B. Exercise training and endothelial function in type 2 diabetes versus non-diabetes						
Allen et al. [31]; USA	66	27	Diabetics: 30–40 min/session of walking, with intensities gradually increased from the one set to the workload resulting in claudication pain during maximal treadmill test, 3 times/week	12	240 mmHg	NA
	69	25	Non-diabetics: the same as diabetics			
Madsen et al. [32]; Denmark	56	31.1	Diabetics: 20 min/session of cycling consisted of 10 intervals of 1 min exercise at 65–69% HR_max_ with a 1 min active recovery exercise, 3 times/week	8	≥220 mmHg	NA
	52	30.5	Non-diabetics: the same as diabetics			
Schreuder et al. [33]; Netherlands	59	32.4	Diabetics: 55 min/session of a circuit of resistance exercises (six exercises, 3 series of 12 repetitions for each exercise) interspersed with aerobic exercise (cycling/running, 5 min for each) at 70–75% HRR, with intensities gradually increased, 3 times/week	8	220 mmHg	NA
	58	26.9	Non-diabetics: the same as diabetics			

*BMI* body mass index, *FMD* flow-mediated dilation, *VO*_*2peak*_ peak oxygen uptake, *HR*_*max*_ maximum heart rate, *MVC* maximal voluntary contraction, *HRR* heart rate reserve, *NA* not applicable

^a^ They represented the baseline mean data for each group

^b^ It represented the results on the comparisons between intervention and control groups

^c^ Both studies allowed for three independent comparisons for each [17, 18]

Of the 16 studies included, the durations of exercise interventions ranged from 8 to 26 weeks, with the majority being 12 weeks; and the frequency of exercise varied from 3 to 5 times/week. The intensity for aerobic exercise was moderate to vigorous in general based on the position statement on physical activity and exercise intensity by Norton and colleagues [34]. The time for one session of aerobic exercise ranged from 20 to 60 min. For resistance exercise, it was composed of 2–3 sets ranging from 10 to 15 repetitions; however, its intensity was generally not well defined except three reported to be 50–70% of one repetition maximum. There was one study adding a moderate energy-restricted dietary program [19] and another one adding standard care [22] to both the intervention and control groups.

The approaches for assessing FMD were well described in each individual study except the one by Hollekim-Strand and colleagues [24]. All participants were required to be fasted for measuring FMD. The cuff was frequently placed at the upper-arm in FMD measurement procedures, with inflated cuff pressure ranging from 30 to 50 mmHg above the systolic blood pressure or from 200 to 250 mmHg to occlude the brachial artery for 4.5–5 min (Table 1). The images of post-deflation diameter were continuously recorded within a time-window that logged from the last 30 s of occlusion through 180 s of hyperemia. Of the included studies, four reported the approaches for randomization, and three utilized per-protocol analyses (see Additional file 1: Table S2). The dropout rates of included studies were low in general except two around 20%.

### Exercise training and endothelial function in type 2 diabetes

#### Compared with non-exercise controls

**Fig. 2:**
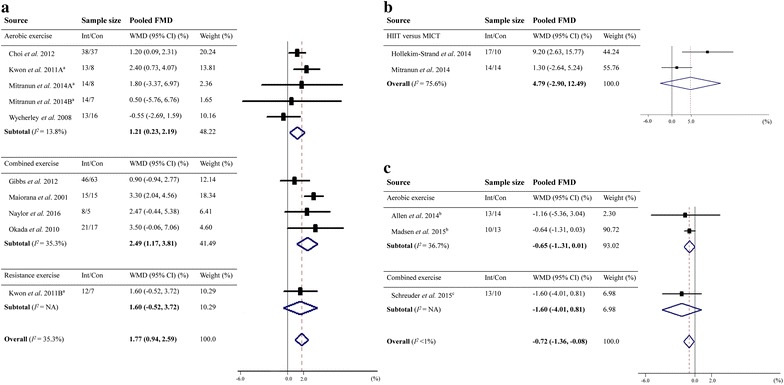
Pooled estimates of the effects of exercise training on FMD among type 2 diabetes patients or between type 2 diabetics versus non-diabetics. **a** Meta-analysis of the exercise training effects on FMD in type 2 diabetes patients compared with non-exercise controls. **b** Meta-analysis of the exercise training effects on FMD in type 2 diabetes patients across different exercise types. **c** Meta-analysis of the exercise training effects on FMD in type 2 diabetics versus non-diabetics. *FMD* flow-mediated dilation, *Int* intervention, *Con* control, *WMD* weighted mean difference, *CI* confidence interval, *HIIT* high-intensity interval training, *MICT* moderate-intensity continuous training, *NA* not applicable. ^a^ Both studies allowed for two independent comparisons for each versus non-exercise controls [17, 18]. ^b^ Standard deviations were obtained using the transformations from *t* and *P* values for differences in means according to the methods suggested in Cochrane Handbook for Systematic Reviews [29]. ^c^ Change-from-baseline standard deviations were obtained using a correlation coefficient of 0.50 according to the methods suggested in Cochrane Handbook for Systematic Reviews [29]

Ten RCTs from eight studies enrolling 377 patients with type 2 diabetes were included [16–23]. Meta-analysis showed that exercise training led to an overall improvement in FMD by 1.77% (95% CI 0.94–2.59%), with no evidence of heterogeneity (*I*^*2*^ = 35.3%; Fig. 2a). When taking exercise types into consideration, results showed that the FMD was increased by 1.21% (95% CI 0.23–2.19%, *I*^*2*^ = 13.8%) for aerobic exercise (five RCTs with 168 patients), 2.49% (95% CI 1.17–3.81%, *I*^*2*^ = 35.3%) for combined aerobic and resistance exercise (four RCTs with 190 patients), and 1.60% (95% CI − 0.52 to 3.72%) for resistance exercise (one RCT with 19 patients).

Univariate meta-regression analyses suggested that changes in FMD related to exercise training or specifically aerobic or combined exercise was not significantly associated with any of the baseline values or changes scores of BMI, blood pressure, glycemic control, or peak oxygen consumption (all *P* > 0.05; Table 2). Sensitivity analyses indicated that the improvements in FMD associated with exercise training or specifically aerobic or combined exercise still remained significant and were not significantly altered after excluding studies having non-exercise interventions, reporting substantial changes in medication use, or utilizing the per-protocol analyses (Table 3). No evidence of publication bias was observed for FMD results associated with exercise training or specifically aerobic or combined exercise (All *P* > 0.80 for both Begg’s and Egger’s tests).

**Table 2:** Meta-regression analyses on FMD in patients with type 2 diabetes

Variables	All exercise			Aerobic exercise			Combined exercise		
	N	*β*	*P*	N	*β*	*P*	N	*β*	*P*
Age (log-transformed)^a^	10	− 0.53	0.72	5	20.1	0.17	4	− 0.06	0.97
Disease duration^a^	6	− 0.03	0.82	3	− 0.08	0.69	2	NA	NA
Training duration	10	− 0.10	0.26	5	NA	NA	4	− 0.13	0.17
Proportion of males^a^	10	0.01	0.50	5	− 0.04	0.20	4	0.06	0.28
Baseline data^a^									
FMD at baseline	9	0.43	0.23	5	1.33	0.29	3	1.16	0.48
Body mass index	10	− 0.16	0.34	5	− 0.30	0.19	4	− 0.32	0.34
Systolic blood pressure	6	0.03	0.70	4	− 0.10	0.34	2	NA	NA
Diastolic blood pressure	6	0.01	0.95	4	0.41	0.30	2	NA	NA
Mean arterial pressure	7	0.23	0.05	4	− 0.19	0.76	3	0.20	0.28
Fasting blood glucose	6	0.17	0.73	4	− 0.71	0.29	2	NA	NA
Hemoglobin A1c	10	1.11	0.09	5	− 1.97	0.44	4	1.21	0.17
Peak oxygen consumption	8	− 0.01	0.95	3	− 3.27	0.61	4	− 0.03	0.95
Change scores post-exercise intervention^b^									
Body mass index	6	− 2.10	0.55	3	− 4.64	0.57	3	3.60	0.59
Systolic blood pressure	6	− 0.16	0.30	4	− 0.20	0.28	2	NA	NA
Diastolic blood pressure	6	− 0.10	0.55	4	− 0.22	0.35	2	NA	NA
Fasting blood glucose	5	1.44	0.62	4	− 4.66	0.34	1	NA	NA
Hemoglobin A1c	9	− 2.52	0.24	5	− 5.02	0.12	3	1.96	0.73
Peak oxygen consumption	6	− 0.38	0.74	3	1.14	0.55	3	− 1.40	0.52

*FMD* flow-mediated dilation, *NA* not applicable

^a^ They were the baseline mean values of intervention and control groups

^b^ They were the average change cores from baseline between intervention and control groups except the study by Maiorana and colleague [21]

**Table 3:** Sensitivity analyses on FMD in patients with type 2 diabetes

Study characteristics	All exercise			Aerobic exercise			Combined exercise		
	N	WMD (%)	95% CI	N	WMD (%)	95% CI	N	WMD (%)	95% CI
Exercise training as the sole intervention	8	2.00	1.23–2.77%	4	1.64	0.79–2.49%	3	2.47	0.70–4.23%
No or minor changes in medication use	8	2.16	1.48–2.85%	4	1.64	0.79–2.49%	3	3.20	2.10–4.30%
Using intention-to-treat analysis	8	2.39	1.64–3.15%	3	2.27	0.94–3.61%	4	2.49	1.17–3.81%

*FMD* flow-mediated dilation, *WMD* weighted mean difference, *CI* confidence interval

#### Compared across exercise types

Three RCTs made comparisons between the effects of different exercise types on FMD in patients with type 2 diabetes [17, 18, 24]. Results showed that neither high-intensity interval aerobic exercise significantly improved FMD over moderate-intensity continuous exercise (two RCTs, WMD 4.79%, 95% CI − 2.90 to 12.49%; Fig. 2b), nor aerobic versus resistance exercise (one RCT, WMD 0.80%, 95% CI − 1.09 to 2.69%).

### Exercise training and endothelial function in type 2 diabetics vs non-diabetics

Three controlled studies with 36 type 2 diabetics and 37 non-diabetics were included in this meta-analysis [31–33]. In these studies, increases in FMD were observed among type 2 diabetics (from 0.5 to 0.82%) and non-diabetics (from 1.46 to 2.1%) following 8–12 weeks of exercise training. Pooled results indicated that the magnitude of the improvements in FMD was smaller in type 2 diabetics than non-diabetics after exercise training (WMD − 0.72%, 95% CI − 1.36 to − 0.08%; *I*^*2*^ < 1%; Fig. 2c) or specifically, after aerobic exercise (two studies, WMD − 0.65%, 95% CI − 1.31 to 0.01%; *I*^*2*^ = 36.7%).

## Discussion

### Summary of the main findings

Our meta-analysis revealed that exercise training, in particular aerobic and combined aerobic and resistance exercise, significantly improved endothelial function in patients with type 2 diabetes, as indicated by increased FMD; and this manner seemed to be independent of changes in traditional cardiometabolic markers including BMI, blood pressure, glycemic control, or cardiorespiratory fitness in relation to exercise training. However, our meta-analysis did not provide adequate evidence that high-intensity interval aerobic exercise was superior to moderate-intensity continuous aerobic exercise in improving endothelial function. Noteworthy, the increases in FMD in response to exercise training in type 2 diabetics were lower than that in non-diabetics, indicating that the presence of diabetes may weaken the exercise training effects on endothelial function.

### Interpretations

Our study showed that exercise training, a non-pharmacological therapy, led to an overall improvement in FMD by 1.77%. This is of clinical importance for patients with type 2 diabetes, since on the one hand, every 1% increase in FMD was correlated with an estimated 13% risk reduction of cardiovascular events based on the report from van Sloten and colleagues [35]; and on the other hand, such an increase in FMD is even larger than or comparable to those from pharmacological interventions like statin therapy [36] or phosphodiesterase inhibitors use [37], which result in an improvement in FMD by 0.94% (95% CI 0.38–1.5%) and 2.19% (95% CI 0.48–3.90%), respectively. Although not fully understood, it is speculated that the observed beneficial effects of exercise training on endothelial function might be underlined by the following mechanisms. Firstly, exercise training causes an increase in blood flow, which augments shear stresses on the endothelium, leading to increased nitric oxide synthesis and bioavailability [1]. Secondly, exercise training reduces oxidative stress and the expression of pro-inflammatory molecules [38], both of which are considered initiating factors for endothelial dysfunction. Thirdly, exercise training may help to restore the function of endothelial progenitor cells, promoting endothelial repair and facilitating vascular angiogenesis subsequently [39].

Partly in agreement with our finding, the prior meta-analysis by Montero and colleagues as well as the review paper by Miele and colleagues had also observed the beneficial effects of exercise training on endothelial function assessed by FMD in patients with type 2 diabetes [13, 26]. However, our study, which included more RCTs and introduced subgroup and meta-regression analyses, extended their findings by providing in-depth analyses of the training effects from the specific exercise type on FMD and exploring the potential moderators. In our study despite a non-significant increase in FMD after resistance exercise, both aerobic and combined exercise were effective in improving FMD, which corresponds well with the results from the meta-analysis conducted in a heterogeneous adult population [40]. Yet inconsistent with our finding, Way and colleagues argued that aerobic exercise may not be able to improve FMD in patients with type 2 diabetes [41], which might be due to their limited number of studies included.

In addition, the largest increase in FMD observed in combined exercise suggests that this mixed form might be superior to aerobic or resistance exercise in improving endothelial function based on our subgroup analyses across exercise types with indirect comparisons. However, it is noteworthy that they may not control for energy expenditure or training duration in every section, and that there was only a single study with a small sample size that explored the influence of resistance exercise on endothelial function [17], which might affect the outcomes of interest (e.g., may underestimate the effects of resistance exercise). Future studies are in need to determine which exercise type might be the best one in increasing FMD using head-to-head designs with the energy expenditure- and/or training time-matched for each section across different exercise types.

In recent years Ramos and colleagues reported that high-intensity interval aerobic exercise, which acts in a time-saving manner [42, 43], produces a greater positive influence on endothelial function versus moderate-intensity continuous aerobic exercise in a mixed adult population [44]. However, our meta-analysis in patients with type 2 diabetes did not provide adequate evidence in support of this notion, which might be largely attributable to the differences in the target populations as well as the small number of studies included. Moreover, Ramos and colleagues pointed out that the improvement in endothelial function associated with high-intensity interval aerobic exercise over moderate-intensity continuous exercise might be owing to its superiority in increasing cardiorespiratory fitness, improving glycemic control, and lowering blood pressure, suggesting a potential positive linkage between endothelial function and cardiometabolic markers [44]. Yet our meta-regression analyses, based on the averages of participant characteristics for each study did not support such an assumption, which could be also evidenced by the findings from the individual study by Gibbs and colleagues [20] and the cross-sectional observation [45]. It seems likely that the benefits of exercise training on endothelial function are independent of improvements in cardiometabolic health among patients with type 2 diabetes, which, albeit, still requires further investigations using the individual participant data.

In addition to suggesting a positive influence of exercise training on endothelial function in patients with type 2 diabetes, our study showed that the improvement in endothelial function in response to chronic exercise training was weakened in patients with type 2 diabetes compared with those without. Although the exact mechanism is not well understood, it is speculated that the presence of diabetes may compromise the ability of the endothelium to endogenously increase vascular nitric oxide bioavailability following exercise training [31], possibly because of the persistent hyperglycemia and the increased levels of circulating advanced glycation end products or reactive oxygen species [46, 47]. Moreover, the blunted potential of exercise to stimulate and to mobilize endothelial progenitor cells observed in diabetic patients compared with non-diabetic controls might also contribute to the weakened improvement in endothelial function in response to exercise among type 2 diabetes [46]. These may indicate that more interventions apart from promoting physical exercise might be needed for patients with type 2 diabetes to obtain comparable health benefits like healthy or non-diabetic controls.

### Limitations

Despite a comprehensive exploration of the exercise training effects on endothelial function among patients with type 2 diabetes, this meta-analysis has some limitations. Firstly, some studies reported changes in medication use in the intervention period or used co-interventions like dietary programs [48], which may influence the endothelial function and contributed to heterogeneity. However, our sensitivity analyses indicated that they are unlikely to have important impacts on the main findings. Secondly, most of the included studies implemented exercise interventions within 12 weeks, which largely represents a relatively short-term effect on endothelial function, although our meta-regression analysis suggested that intervention periods were not likely to affect the changes in endothelial function associated with exercise training. More studies are therefore warranted to explore the long-term (e.g., over 6 months) effects of exercise training on endothelial function, just as suggested by Lenasi and colleagues [46]. Thirdly, some variances existed in the measurement methods for FMD. However, these methods were well defined in general. Although it is reported that the measurement of peak dilation at 60 s after cuff release may result in an underestimation of true FMD by up to 40% [49], this factor seems to have had a minor influence on our outcomes on FMD since all studies except the one by Hollekim-Strand and colleagues [24] had already adopted a continuous measurement of post-deflation diameter within the time-window as aforementioned. Finally, our study did not search for unpublished studies and had a language restriction, which may cause some selection bias.

## Conclusion

In summary, this meta-analysis indicates that exercise training, especially aerobic or combined aerobic and resistance exercise, improves endothelial function in patients with type 2 diabetes. Such an improvement is likely to be independent of changes in traditional cardiometabolic markers associated with exercise training, but appears to be weakened compared with a non-diabetes state. Notably, despite a larger effect that was seen with combined exercise in improving endothelial function compared with aerobic or resistance exercise alone, studies did not have specifications on the controls for energy expenditure or training time for every section. Future studies with longer intervention durations are required to sort out the optimal exercise type to improve pathological conditions of endothelial dysfunction in patients with type 2 diabetes using head-to-head designs with the energy expenditure- and/or training time-matched for each section across different exercise types.

## Additional file

**Additional file 1: Table S1.** Search strategies. **Table S2.** Quality assessment.

## Abbreviations

RCTs: randomized controlled trials
FMD: flow-mediated dilation
PRISMA: Preferred Reporting Items for Systematic Reviews and Meta-Analyses
BMI: body mass index
CIs: confidence intervals
WMD: weighted mean difference
