# Supplementary material for: Exercise training and endothelial function in patients with type 2 diabetes: a meta-analysis
Source: Cardiovasc Diabetol. 2018 May 2;17:64. doi: 10.1186/s12933-018-0711-2 (PMC5930739; doi:10.1186/s12933-018-0711-2)
Supplement: Supplementary file 1 — Additional file 1: Table S1. Search strategies. Table S2. Quality assessment. [file 12933_2018_711_MOESM1_ESM.docx]

# Table S1. Search strategies.

**Search strategy in PubMed**

#1 endothelium[Text Word] OR endothelium[Title/Abstract]) OR vascular function[Text Word] OR vascular function[Title/Abstract] OR endothelial[Text Word] OR endothelial[Title/Abstract] OR flow-mediated dilation[Text Word] OR flow-mediated dilation[Title/Abstract] OR flow mediated dilation[Text Word] OR flow mediated dilation[Title/Abstract] OR endothelial function[Text Word] OR endothelial function[Title/Abstract] OR FMD[Text Word] OR FMD[Title/Abstract] OR artery blood flow[Text Word] OR artery blood flow[Title/Abstract]

#2 exercise[Title/Abstract] OR exercise[Text Word] OR training[Title/Abstract] OR training[Text Word] OR physical activity[Title/Abstract] OR physical activity[Text Word] OR sport*[Title/Abstract] OR sport*[Text Word]

#3 diabet*[Text Word] OR diabet*[Title/Abstract] OR diabetes mellitus, type 2[MeSH Terms]

#4 #1 AND #2 AND #3

#5 #4 Filters: English, published up to January 12^nd^, 2018

**Search strategy in the Cochrane Central Register of Controlled Trials**

#1 endothelium[Title/Abstract/Key Word] OR vascular function[Title/Abstract/Key Word] OR endothelial[Title/Abstract/Key Word] OR flow-mediated dilation[Title/Abstract/Key Word] OR flow mediated dilation[Title/Abstract/Key Word] OR endothelial function[Title/Abstract/Key Word] OR FMD[Title/Abstract/Key Word] OR artery blood flow[Title/Abstract/Key Word]

#2 exercise[Title/Abstract/Key Word] OR physical activity[Title/Abstract/Key Word] OR sport*[Title/Abstract/Key Word]

#3 diabet* [Title/Abstract/Key Word] diabetes mellitus, type 2 [MeSH Terms]

#4 #1 AND #2 AND #3

#5 #4 Filters: published up to January 12^nd^, 2018 (by hand)

**Search strategy in Web of Science**

#1 endothelium[Topic] OR vascular function[Topic] OR endothelial[Topic] OR flow-mediated dilation[Topic] OR flow mediated dilation[Topic] OR endothelial function[Topic] OR FMD[Topic] OR artery blood flow[Topic]

#2 exercise[Topic] OR physical activity[Topic] OR sport*[Topic] OR training[Topic]

#3 diabet*[Topic]

#4 #1 AND #2 AND #3

#5 #4 Filters: References Types (article or other or clinical trial or review); published up to January 12^nd^, 2018 (by hand)

# Table S2. Quality assessment.

| Author, year | Random sequence generation | Allocation concealment | Blinding of participants and personnel^a^ | Blinding of outcome assessment^b^ | Incomplete outcome data addressed | Selective reporting |
| --- | --- | --- | --- | --- | --- | --- |
| Choi *et al.* 2012 | Low | Unclear | Low | Low | High | Low |
| Kwon *et al.* 2011^c^ | Unclear | Unclear | Low | Low | Low | Low |
| Mitranun *et al.* 2014^c^ | Unclear | Unclear | Low | Low | Low | Low |
| Wycherley *et al.* 2008 | Unclear | Unclear | Low | Low | High | Low |
| Hollekim-Strand *et al.* 2014 | Low | Unclear | Low | Low | High | Low |
| Gibbs *et al.* 2012 | Unclear | Unclear | Low | Low | Low | Low |
| Maiorana *et al.* 2001 | Unclear | Unclear | Low | Low | Low | Low |
| Naylor *et al.* 2016 | Low | Unclear | Low | Low | Low | Low |
| Okada *et al.* 2010 | Low | Unclear | Low | Low | Low | Low |
| Allen *et al.* 2014 | NA | Unclear | Low | Low | Low | Low |
| Madsen *et al.* 2015 | NA | Unclear | Low | Low | Low | Low |
| Schreuder *et al.* 2015 | NA | Unclear | Low | Low | Low | Low |

^a^ Because these studies were aimed to investigate the effects of exercise training, complete blinding of participants and personnel is impossible and unnecessary.

^b^ The outcome assessment for flow-mediated dilation is objective, which is not likely to be affected by lacking of blinding.

^c^ Both studies allowed for 3 independent comparisons for each.
